# Glenohumeral relationship in maximum elevation

**DOI:** 10.1007/s00276-014-1257-y

**Published:** 2014-01-24

**Authors:** Hiroaki Inui, Katsuya Nobuhara

**Affiliations:** Nobuhara Hospital and Institute of Biomechanics, 720 Haze, Issaicho, Tatsunoshi, Hyogo 679-4017 Japan

**Keywords:** Shoulder, Glenohumeral joint, Maximum elevation, External rotation, MRI

## Abstract

**Purpose:**

The purpose of this study was to clarify rotational relationships between the anatomical landmarks of the glenohumeral joint in maximum elevation.

**Methods:**

Twenty-five healthy volunteers (20 men, 5 women; mean age, 31 years) held the arm in maximum elevation in an open MRI system. In each three-dimensionally computer-generated image, elevation angle of the humerus in the plane of elevation was measured, based on the glenoid and the scapular planes. Using the equator set on the head surface by the plane parallel to the humeral axis, involving the head center and the bicipital groove, glenoid location and rotational relationships were investigated.

**Results:**

The elevation angle was 102° ± 9° in the plane 7° ± 8° anterior to the scapular plane, and axial rotation was fixed with the glenoidal long axis parallel to the equator (within 2°). Each glenoid center located on antero-superior portion of the humeral head, and the direction from the top of the head to its location was the same as that of the shaft tilting, indicating the glenoid only translated without rotation after reaching the top of the head on the equator.

**Conclusions:**

Before reaching maximum elevation, the glenohumeral joint would be locked in axial rotation. The position when the glenoid is on the top of the humeral head with the humeral shaft perpendicular to the glenoid is considered to be essentially the final position of elevation, above which the glenohumeral joint only translates without axial rotation even if the humerus is more elevated.

**Electronic supplementary material:**

The online version of this article (doi:10.1007/s00276-014-1257-y) contains supplementary material, which is available to authorized users.

## Introduction

Arm elevation is one of the most important functions of the shoulder in our daily lives [4, 12, 13]. Many studies [1, 3, 5, 7, 9, 11, 14, 16, 17] approached the topics about arm elevation, one [11] of which indicated that the humerus moves toward a similar final position at the end of raising the arm irrespective of the plane of elevation, and its position is one of external rotation and elevation of the humerus relative to the scapula, slightly anterior to the plane of the scapula. However, they only measured rotational angles of the glenohumeral joint and could not clarify the relationships between the anatomical landmarks of its joint. When the arm reaches its position, most proximal part of the humerus is covered with the acromion, making it difficult to know the anatomical position of the joint. We investigated rotational relationships between the anatomical landmarks of the glenohumeral joint in maximum elevation.

## Materials and methods

**Fig. 1:**
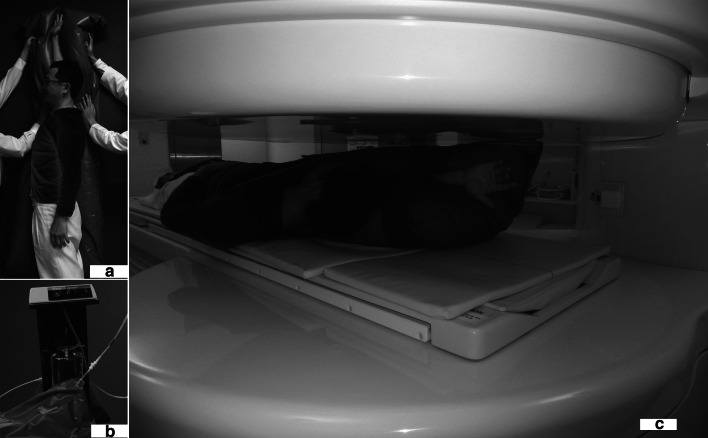
**a** The arm was placed in maximum elevation in which the tips of fingers reached highest in a standing position. A surgical mattress was adopted around his upper extremity and body to reproduce the position. **b** The mattress is made of micro-balls of styrofoam, which are stabilized by vacuum. **c** Volunteer with the mattress, lying in oblique supine position in an open MRI (Magnetom Open, Siemens, Germany)

Twenty-five volunteers (20 men, 5 women) without symptoms or history of shoulder disease were enrolled in the study. Mean age was 29 (21–35) years. All participants provided informed consent. The maximum elevation of the shoulder was defined as the position in which the tips of fingers could reach highest in a standing position. A surgical mattress (VACUFORM; Schmidt, Garbsen, Germany) made of micro-balls of styrofoam, which could be stabilized by vacuum, was adopted around his or her upper extremity and body to reproduce shoulder position (Fig. 1). The amount of pronation or supination of the forearm was not specified. Each participant maintained the same maximum elevation with that surgical mattress, lying supine in a 0.2-T MRI system (Magnetom Open, Siemens, Munich, Germany). The shoulders were imaged using a three-dimensional (3D) gradient echo (repetition time, 56 ms; echo time, 25 ms; flip angle, 40°) with 2-mm thickness. All images were obtained with an 18-cm FOV and a 256 × 192 matrix. Imaging process required an average of 10 min.

All data were transferred to a computer (O2; SGI, Mountain View, CA) and a 3D image of the glenohumeral joint including the proximal part of the humerus was generated using computer software (3D-Virtuoso, Siemens). Providing instant access to 3D information, the same software allowed anatomies to be viewed from any angle.

**Fig. 2:**
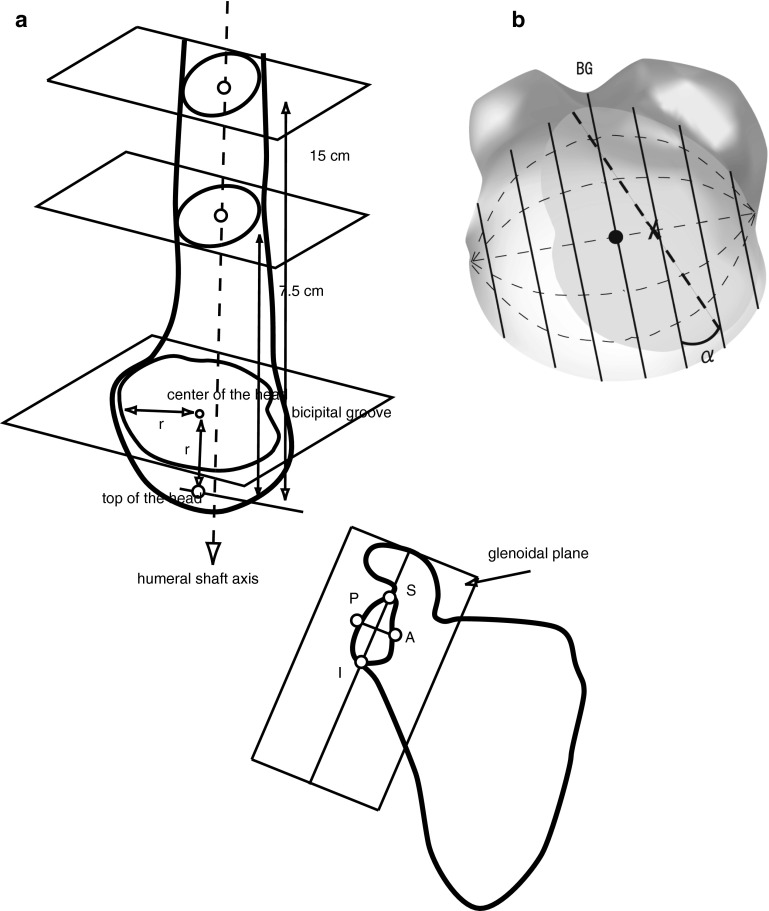
**a** Illustrations showing the anatomical landmarks such as glenoidal long axis (the* line* between superior and inferior rims), glenoidal transverse axis (the* line* between anterior and posterior rims), humeral head center, and humeral shaft axis. *A* anterior rim, *P* posterior rim, *S* superior rim, *I* inferior rim, *r* humeral radius. The computer screen display shows the long and the transverse axes of the glenoid, the humeral shaft axis, and the humeral center. **b** Global diagram set on the head surface with the plane including the head center (*black dot*) and the bicipital groove, and the parallel planes analogous to latitudes. *Straight lines* represent circles of latitude and *curved broken lines* represent circles of longitude. Rotation (α) is referenced to latitude by rotating the globe to align longitude including the midpoint (*X*) of the glenoidal long axis (*straight broken line*) with the vertical. *BG* bicipital groove

Anatomical landmarks such as the glenoidal long axis, glenoid center, humeral head center, and humeral shaft axis (Fig. 2) were defined as described below [10]. On the glenoid rim, the point just posterior to the coracoid base was defined as the superior rim and the point just anterior to the lateral border of the scapula was defined as the inferior rim. The line connecting these points was defined as the glenoidal long axis. The glenoid plane was defined as the plane including the glenoidal long axis, and was parallel to the transverse axis connecting the anterior and posterior rims on a cross-section at the center level of the glenoid. The scapular plane was defined as the plane including the glenoidal long axis and was perpendicular to the glenoid plane.

Two cross-sections of the humerus were obtained at 7.5 and 15 cm from the proximal end. The center for these cross-sections of the cortical bone was determined by fitting a circle, and the humeral axis was defined as the line that passed through the center of these circles. Using the data of Iannotti et al. [8], which showed correlations between size of the glenoid and the radius of curvature of the humeral head, each humeral radius was calculated as follows: radius (mm) = 24 × length of glenoidal long axis/39 (where 24 is the average head radius and 39 is the average glenoidal long axis in the population as measured by Iannotti et al.). The head was cut in a plane perpendicular to the humeral axis at the distance of the radius from the proximal end, and the center determined by fitting a circle of the same radius. This was regarded as the center of the head. In this plane, the bottom of the bicipital groove was also plotted.

The slope of the humeral long axis on the glenoid was determined by measuring the angle relative to the glenoidal long axis and analyzing their orientation in the transverse plane, relative to the scapular plane, in which the humerus was elevated. Rotation of the glenohumeral joint was visualized on the computer screen as follows. The equator was set on the head surface by the plane parallel to the humeral long axis, including the head center and the bicipital groove. Parallel lines to the equator were analogous to latitudes. Rotation was referenced to latitude by rotating the globe to align the longitude, including the midpoint of the glenoidal long axis, with the vertical. The angle when the glenoidal long axis became parallel to latitude was defined as 0° and values in external rotation were defined as positive.

To investigate location of the glenoid center, the surface of the humeral head was divided into four segments (anterior–superior portion; Zone I, posterior–superior portion; Zone II, anterior–inferior portion; Zone III, and posterior–inferior portion; Zone IV), using the equator and the circle of longitude crossing the top of the head. Meanwhile, the shaft axis and the center of the humerus in each subject were projected orthogonally to the glenoid plane to confirm the relation between the anatomical landmarks.

Ten different glenoids were analyzed measuring length values of the long and the transverse axes by two independent investigators to determine the inter-observer variability. To measure the intra-observer variability, those values in 10 glenoids were measured twice by the same person. Angle values of the plane including the shaft axis and the head center to the equator on the head surface were also analyzed to determine these variabilities for the humerus. All data are expressed as mean ± SD. To assess these variabilities, the interclass and intraclass correlation coefficients were used (Wilcoxon Signed Ranks test).

## Results

### Variability or reproducibility

Length values of the long and the transverse axes of the glenoid, and angle values of the plane including the shaft axis and the head center to the equator on the head surface were 36.0 ± 3.0 mm, 22.5 ± 2.1 mm, 10.0° ± 8.8°. Those values in ten glenoid and humeral bones showed the variability or the reproducibility was high with a correlation coefficient ranging from 0.73 to 0.98 (Wilcoxon Signed Ranks test) (Table 1).

**Table 1:** Variability or reproducibility

	Mean ± SD	Mean ± SD	Correlation coefficient
Variability			
Length: Glenoidal long axis (mm)	36.2 ± 23.7	36.1 ± 3.5	0.98
Length: Glenoidal transverse axis (mm)	22.6 ± 2.4	23.4 ± 2.7	0.86
Angle^a^ (°)	12.3 ± 9.0	12.5 ± 8.8	0.98
Reproducibility			
Length: Glenoidal long axis (mm)	36.2 ± 3.7	36.3 ± 3.4	0.88
Length: Glenoidal transverse axis (mm)	22.6 ± 2.4	23.9 ± 1.6	0.73
Angle^a^ (°)	12.3 ± 9.0	13.0 ± 8.4	0.95

^a^Angle of the plane including the shaft axis and the head center to the equator

The humeral shaft showed 102° ± 9° of elevation in the plane which faces 7° ± 8° anterior to the scapular plane on the glenoid. Axial rotation was fixed with the glenoidal long axis parallel to the equator (within 2°).

**Fig. 3:**
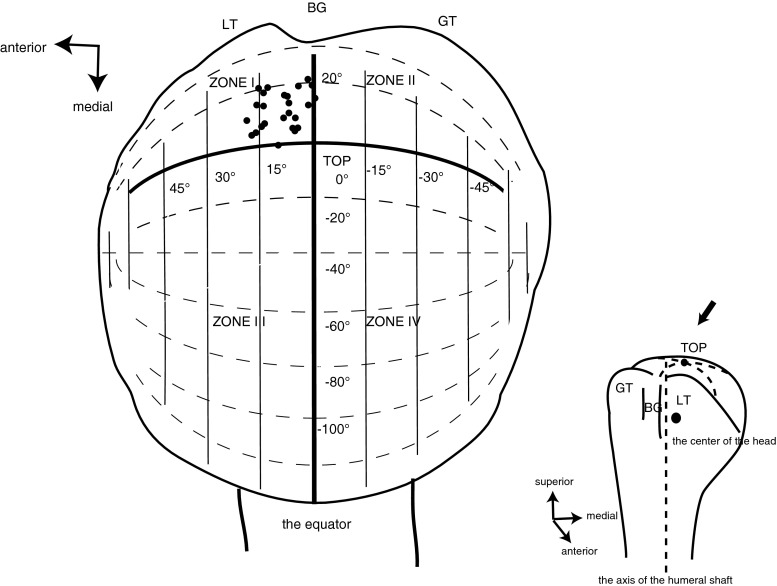
The centers of the glenoid (*black dot*) located on the antero-superior portion of the head. The equator (*solid line*) and the longitude (*solid curved line*) crossing the top on the equator, divide the head surface into four areas. *BG* bicipital groove, *LT* lesser tuberosity, *GT* greater tuberosity

Figure 3 shows which portion of the head surface the center of the glenoid was located on. It showed the glenoid located on the anterior–superior portion (Zone I) of the head in all subjects.

**Fig. 4:**
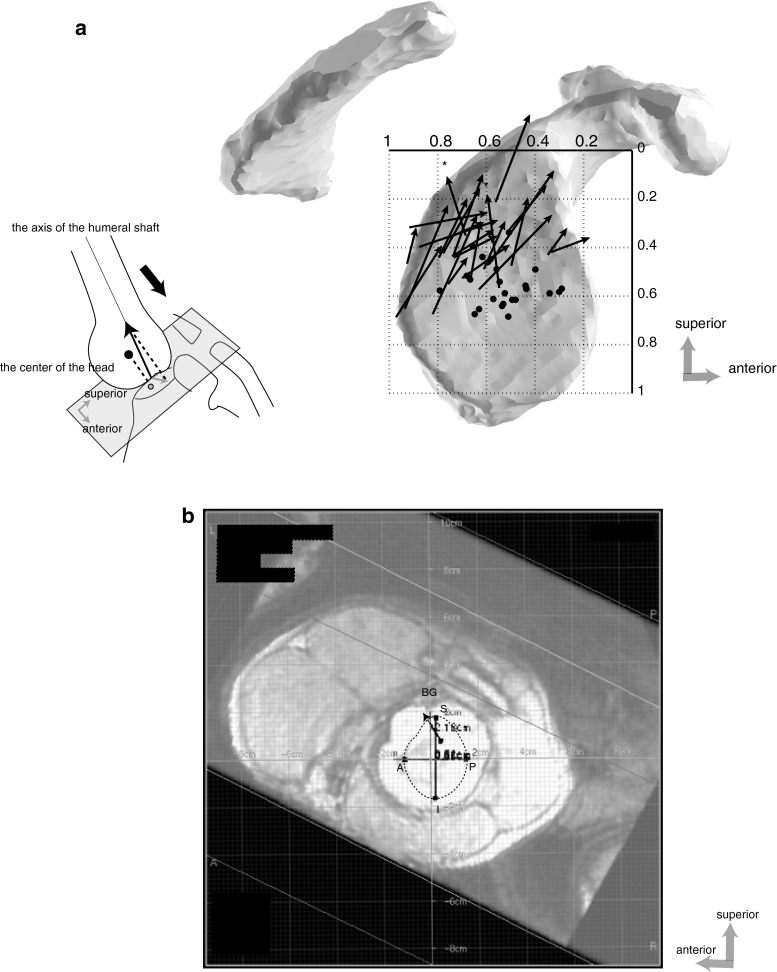
**a** Relationship between the shaft axis and the head center projected on the glenoidal plane is shown. The shaft axes (*black arrow*) except two subjects (*asterisk*) directed obliquely in the antero-superior direction above the head center (*black dot*). The length is dependent on the angle of the shaft tilting on the glenoid, indicating that the shorter is the length of the arrow, the more vertical is the shaft to the glenoid surface. **b** The computer screen display shows the long and the transverse axes of the glenoid, the humeral shaft axis (*black arrow*). The center of the grid on the computer screen consisted with the center of the humeral head which was cut by the plane including its center. *A* anterior rim, *P* posterior rim, *S* superior rim, *I* inferior rim, *BG* bicipital groove

The humeral head centers and shaft axes projected on the glenoidal plane are shown in Fig. 4. The center of the humeral head projects approximately on the center of the glenoid, showing the humeral head remained centered in the glenoid cavity, and the shaft axes were located above these centers. The same length of axis was projected, and the lengths of their shadows were different between the subjects, indicating the angle of the shaft to the glenoid varied. However, the humeral axis tilted in about the same direction (antero-superiorly) above the center of the glenoid. The direction of the shaft tilting was the same as that of translation from the top of the head to each glenoid location as was mentioned above.

The final position was proved to be the same in all subjects, suggesting this position is unique and may be reached whatever the course of the humerus.

## Discussion

The glenohumeral joint was considered to reach a general position at the end of raising the arm whatever the passway followed by the humerus [4, 6, 11, 13]. However, there has been no precise in vivo description of the glenohumeral joint in maximum elevation. Some discrepancies existed in the past studies about the position of the glenohumeral position in maximum elevation. Browne et al. [3] found that the plane of maximum elevation was 23° anterior to the scapular plane, while Pearl et al. [15] reported the same plane was 4° posterior to the scapular plane. One reason for explaining the discrepancies might be that those studies used different methods. The former was a cadaveric study and the latter study used the scapular locating device for estimating locations of the scapula in vivo. Cadaveric studies possess limitations in that the scapula is affixed to a support without outer muscles as well as the clavicle and ribs. On the other hand, locating devices have a problem in estimation of location of the bones. Three-dimensional computer-generated MRI without radiation exposure on subjects would be appropriate for analyzing a static position because relations between the bony landmarks can be investigated through trial and error on a computer screen.

For investigating rotational alignment of the joint, we set the equator and its parallel lines on the surface of the humeral head. The equator was determined to include the bicipital groove because several authors [4, 12, 13] suggested its importance to glenohumeral motion or position. In the result, all subjects showed that the glenoidal long axis became parallel to the equator in maximum elevation. As long as axial rotation in the glenohumeral joint is expressed as rotational angle of the humeral axis, those values possibly are varying. One reason for this variation might be that the head has offset and angle of retroversion depends on each subject as was suggested by angle of the plane in Table 1. To the best of our knowledge, this study was the first representing a concrete description about anatomical relationship of rotation in maximum elevation.

Ranges of rotation would be restricted with arm elevation because insertion of the short rotators approaches the glenoid surface and the capsule surrounding the head also becomes tight. Thus, irrespective of the plane of elevation, the shoulder joint would finally converge to the same, i.e., the preferential position. Gagey et al. [6] described behavior of coracohumeral and inferior glenohumeral ligaments that participate in determining the preferential position. In their experimental study, the axis of the humeral shaft in that position was shown to be perpendicular to the glenoid cavity and it also was shown there still existed lateral rotation of the humerus allowing continuation of elevation as a final phase.

**Fig. 5:**
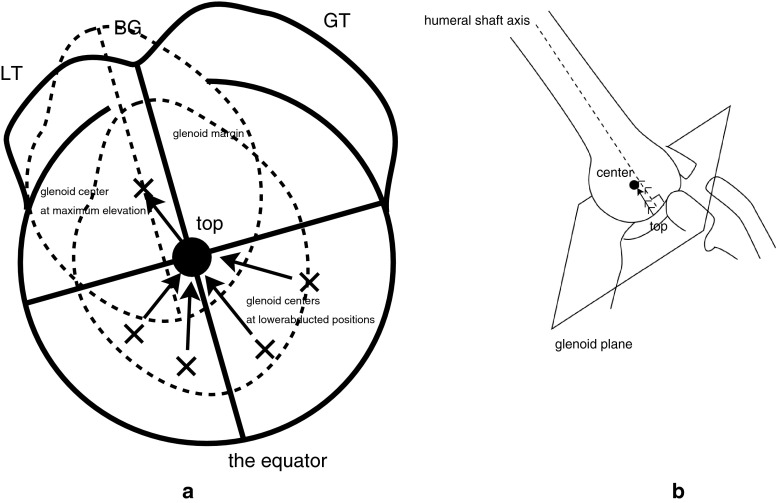
The glenoid center (*X*), locating in the Zones III or IV, moves upward with ranges of rotation getting restricted during the arm elevation. In the figure, four glenoid centers with lower abduction angle are shown. They converge to the top (*black dot*) of the head on the equator with the glenoidal long axis parallel to the equator. At this point the shaft would become perpendicular to the glenoid and the joint might be locked in axial rotation. When the arm is more elevated, the glenoid would only translate to the antero-superior portion (Zone I) of the humeral head without axial rotation

Figure 5 illustrated the trajectory of the glenoid on the humeral head during arm elevation The glenoid moves upward on the surface of the humeral head, converging to the equator with ranges of rotation getting restricted. When the glenoidal long axis becomes parallel to the equator, the glenoid center is located on the top of the head, in which the joint is locked in axial rotation. That position would be essentially the final position of elevation. When the arm is more elevated after that position, the glenoid would only translate to the antero-superior portion of the humeral head without axial rotation (Fig. 5a, b). In fact, the direction of its translation was the same as that of the shaft tilting on the glenoid. Our data supported the studies by Browne et al. [4] showing the plane of maximum elevation was anterior to the plane of the scapula.

Certain shortcomings of this study must be acknowledged. A surgical mattress was adopted around elevated arm and body in a standing position and the angle of the upper extremity to the trunk was reproduced in an open MRI system. However, each participant had to lie in a supine position in the system and it was possible his or her position of the joint was changed. Systems with upright coils should be used for directly investigating the difference in relationship in standing or sitting positions and that would enable determining the effect of gravity on the glenohumeral relationship. We could not clarify which part of anatomy was essential for determining the positioning in maximum elevation in this study. Information about cases with rotator cuff tear or without the long head of the biceps might be helpful for clarifying which part of the joint is essential to determine the positioning. Experiments like ex vivo studies simulating rotator cuff tears [2, 18] might also be useful to know the mechanism around maximum elevation.

Even though those limitations should be taken into consideration, the current study might be sufficient to describe the static position of the glenohumeral joint in maximum elevation. The result of the study suggested (a) the position in which the glenoid is on the top of the humeral head with the humeral shaft perpendicular to the glenoid is essentially the final position of elevation, and (b) the humeral axis is slightly anterior to the plane of the scapula and no axial rotation is available for the humerus after that final position. Rehabilitation programs aiming at reaching this position when the joint is impaired after injury or operation would be reasonable from this biomechanical point of view.

## Electronic supplementary material

Below is the link to the electronic supplementary material.

Supplementary material 1 (WMV 26711 kb)
